# Supplementary material for: Previously uncharacterized rectangular bacterial structures in the dolphin mouth
Source: Nat Commun. 2023 Apr 13;14:2098. doi: 10.1038/s41467-023-37638-y (PMC10102025; doi:10.1038/s41467-023-37638-y)
Supplement: Supplementary file 1 — Supplementary Information [file 41467_2023_37638_MOESM1_ESM.pdf]

SUPPLEMENTARY INFORMATION for "Previously uncharacterized rectangular bacterial structures in the dolphin mouth"

Natasha K. Dudek[1,2,11], Jesus G. Galaz-Montoya[3], Handuo Shi[3,4], Megan Mayer[5,12], Cristina Danita[3], Arianna I. Celis[4], Tobias Viehboeck[6,7], Gong-Her Wu[3], Barry Behr[8], Silvia Bulgheresi[6], Kerwyn Casey Huang[3,4,9], Wah Chiu[3,4,5], David A. Relman[1,4,9,10,*]

[1]Department of Medicine, Stanford University School of Medicine, Stanford, CA 94305, USA

[2]Department of Ecology and Evolutionary Biology, University of California, Santa Cruz, Santa Cruz, CA 95064, USA

[3]Department of Bioengineering, Stanford University, Stanford CA 94305, USA

[4]Department of Microbiology and Immunology, Stanford University School of Medicine, Stanford, CA 94305, USA

[5]Division of CryoEM and Bioimaging, SSRL, SLAC National Accelerator Laboratory, Menlo Park, CA 94025, USA

[6]Department of Functional and Evolutionary Ecology, Environmental Cell Biology Group, University of Vienna, Vienna, Austria

[7]Division of Microbial Ecology, Center for Microbiology and Environmental Systems Science, and Vienna Doctoral School of Ecology and Evolution, University of Vienna, Vienna, Austria

[8]Department of Obstetrics and Gynecology, Stanford University School of Medicine, Stanford, CA 94305, USA

[9]Chan Zuckerberg Biohub, San Francisco, CA 94158, USA

[10]Infectious Diseases Section, Veterans Affairs Palo Alto Health Care System, Palo Alto, CA 94304, USA

[11]Present address: Quantori, Cambridge, MA 02142, USA

[12]Present address: Department of Biological Chemistry and Molecular Pharmacology, Harvard Medical School, Boston, MA 02115, USA

*Correspondence: relman@stanford.edu

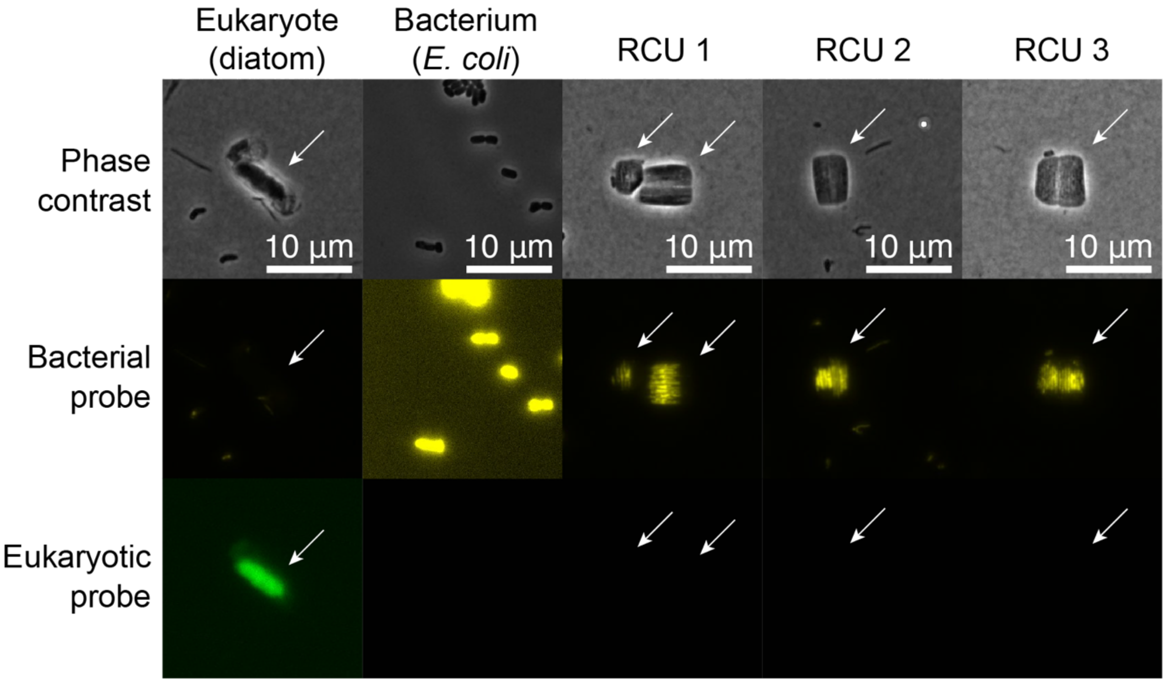

Supplementary Figure 1: Fluorescence *in situ* hybridization indicates that RBSs are bacterial rather than eukaryotic. Bacterial probe Eub-338 was labeled with AlexaFluor-488 and eukaryotic probe Euk-1209 was labeled with AlexaFluor-660. Top: phase-contrast images; middle, bottom: fluorescence images from bacterial and eukaryotic probes, respectively. Arrows indicate the relevant cells in non-axenic samples. The first column is a cell of the marine diatom *Skeletonema costatum* grown in non-axenic culture. The second column is axenic *Escherichia coli* cells. The last three columns are RBSs obtained directly from dolphin oral swabs. The bacterial probe labeled all RBSs, while the eukaryotic probe only labeled the marine diatom.

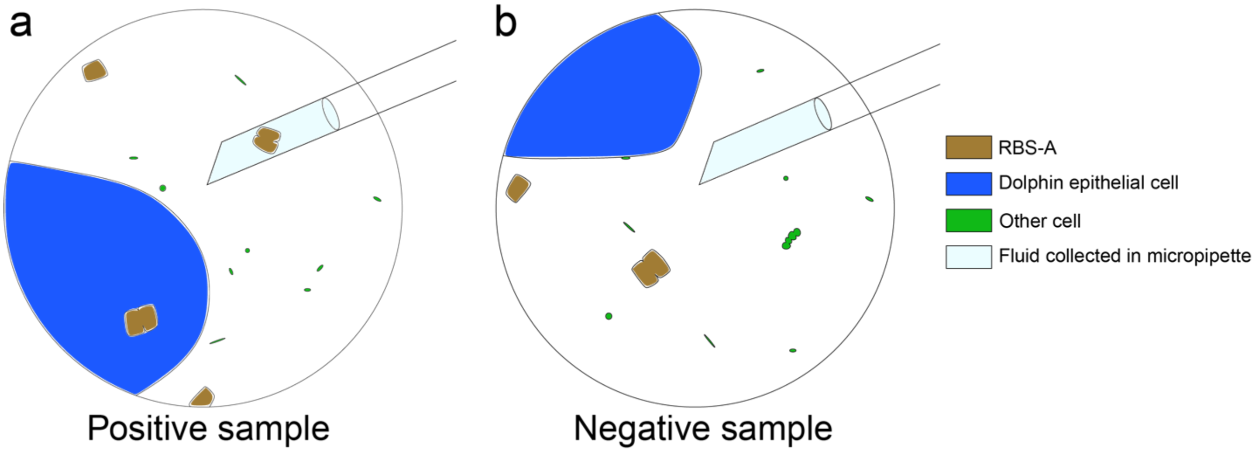

Supplementary Figure 2: Collection strategy for mini-metagenomics. a) Four samples of RBS-As were collected, each with ~1-3 RBS-As per tube. **b)** Four negative control samples with fluid from the dolphin oral sample but not RBS-As were also collected. While care was taken to avoid collecting non-RBS-A cells, it is likely that small, non-visualized cells and/or cell-free DNA were captured along with RBS-As.

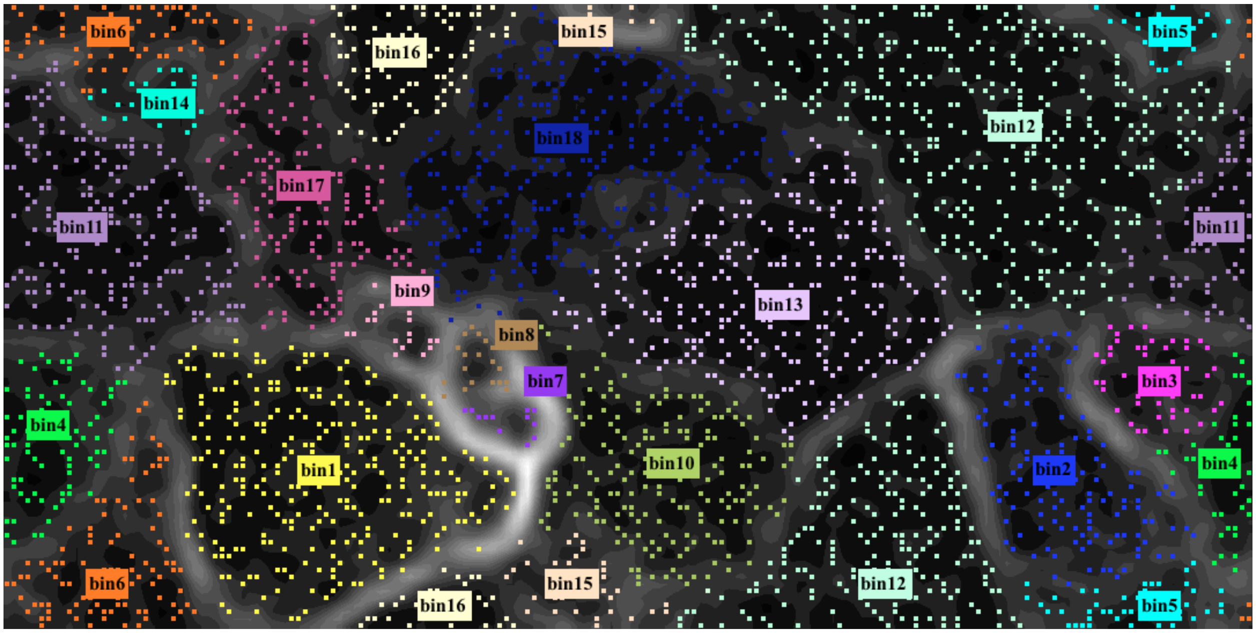

Supplementary Figure 3: Tetranucleotide Emergent Self-Organizing Map (ESOM) used to bin genomes. Scaffolds were split into keys of size 5000. Keys (dots on the ESOM), each of which represents a 5-kb section of scaffold, are color-coded based on the bin to which they were assigned.

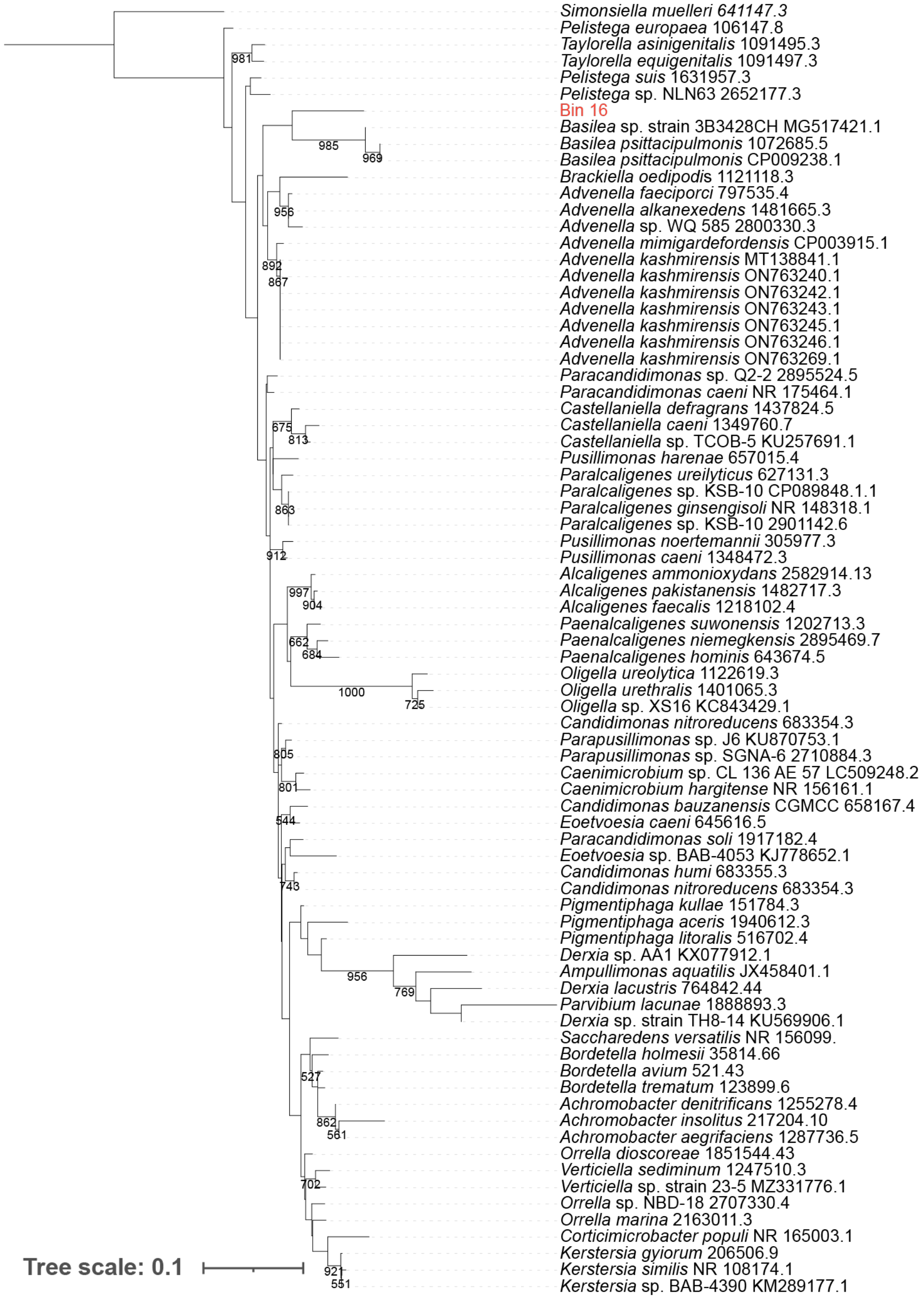

Supplementary Figure 4: Phylogenetic analysis of the 16S rRNA gene confirms that bin 16 is affiliated with the family *Alcaligenaceae*. Maximum likelihood 16S rRNA gene phylogeny of the family *Alcaligenaceae* with 1000 bootstraps. Up to three representative sequences from each genus in the family *Alcaligenaceae*, as listed on the NCBI Taxonomy Browser, were included, as were the top 10 gene sequences most similar to the bin 16 16S rRNA gene as identified through a BLAST[1] query against the NCBI nr/nt database. Bin 16 is highlighted in red; other leaves are represented by

species name and NCBI identifier. Bootstrap support values ≥50% (≥500) are shown. The 16S rRNA gene of *S. muelleri* was used as an outgroup.

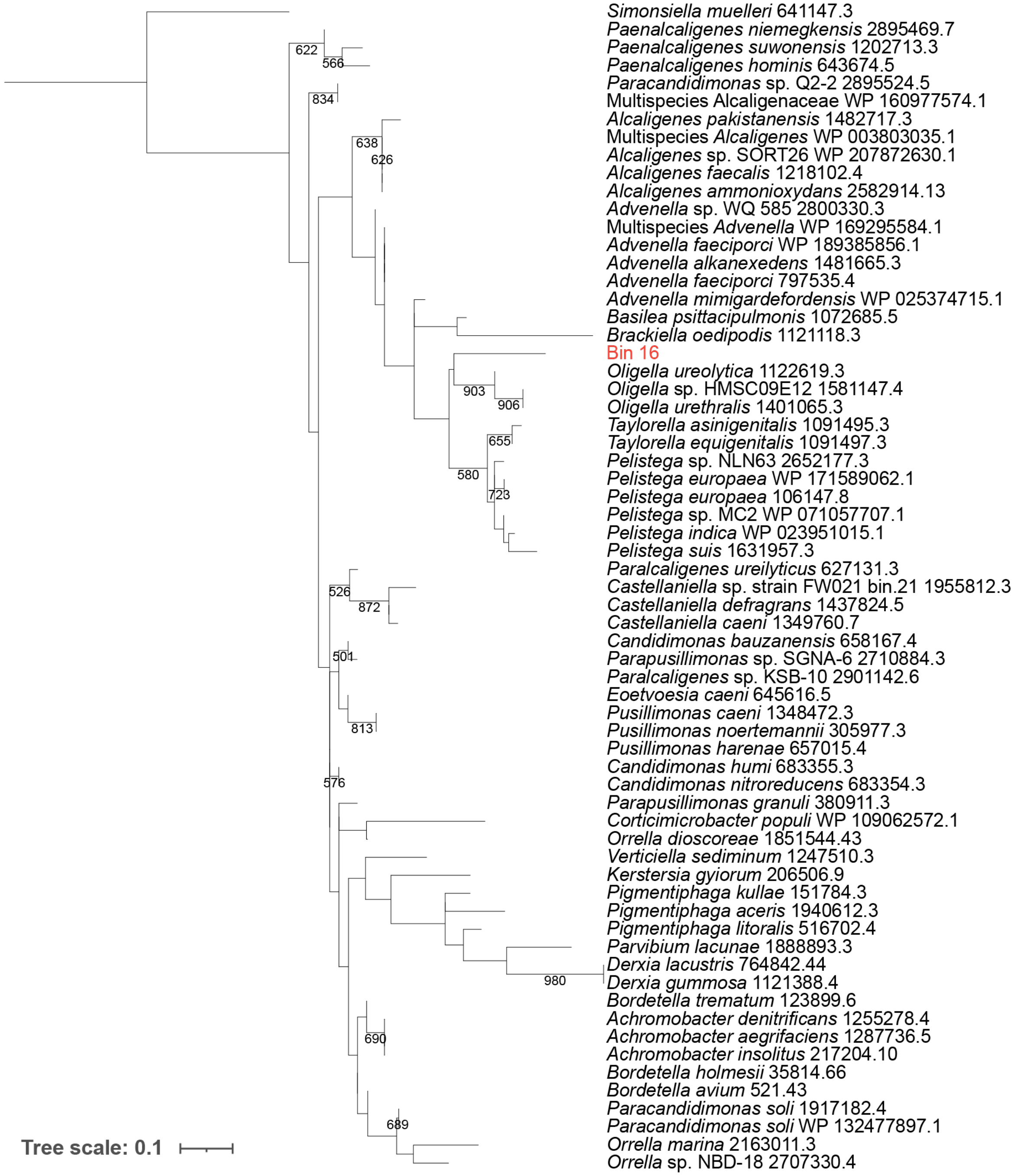

Supplementary Figure 5: Phylogenetic analysis of ribosomal protein S3 (rpS3) confirms bin 16 is affiliated with the family *Alcaligenaceae*. Maximum likelihood rpS3 phylogeny of the family *Alcaligenaceae* with 1000 bootstraps. Up to three representative sequences from each genus in the family *Alcaligenaceae*, as listed on the NCBI Taxonomy Browser, were included, as were the top 10 protein sequences most similar to the bin 16 rpS3 as identified through a BLAST[1] query against the NCBI nr database. Bin 16 is highlighted in red; other leaves are represented by species name

and NCBI identifier. Bootstrap support values ≥50% (≥500) are shown. The rpS3 protein sequence of *S. muelleri* was used as an outgroup.

ASV	# samples	Phylum	Class	Order	Family	Genus	Species
1	13	Proteobacteria	Gammaproteobacteria	Pasteurellales	Pasteurellaceae	*Pasteurella*	*skyensis*
2	13	Proteobacteria	Gammaproteobacteria	Pseudomonadales	Moraxellaceae	-	-
3	13	Proteobacteria	Gammaproteobacteria	Cardiobacteriales	-	-	-
4	13	Proteobacteria	Gammaproteobacteria	Pseudomonadales	Moraxellaceae	-	-
5	13	Bacteroidetes	Flavobacteriia	Flavobacteriales	Flavobacteriaceae	-	-
6	13	Fusobacteriota	Fusobacteriia	Fusobacteriales	Fusobacteriaceae	*Fusobacterium*	-
7	13	Proteobacteria	Gammaproteobacteria	Pasteurellales	Pasteurellaceae	*Pasteurella*	*skyensis*
8	13	Proteobacteria	Epsilonproteobacteria	Campylobacterales	Campylobacteraceae	*Arcobacter*	-
9	13	Proteobacteria	Epsilonproteobacteria	Campylobacterales	Campylobacteraceae	-	-
10	13	Firmicutes	Bacilli	Lactobacillales	Enterococcaceae	*Enterococcus*	-
11	12	Proteobacteria	Gammaproteobacteria	Cardiobacteriales	-	-	-
12	12	Bacteroidetes	Flavobacteriia	Flavobacteriales	Flavobacteriaceae	*Tenacibaculum*	-
13	12	Bacteroidetes	-	-	-	-	-
14	12	Bacteroidetes	-	-	-	-	-
15	12	Bacteroidetes	Flavobacteriia	Flavobacteriales	Flavobacteriaceae	*Tenacibaculum*	-
16	11	Bacteroidetes	Bacteroidia	Bacteroidales	Porphyromonadaceae	*Porphyromonas*	-
17	11	Proteobacteria	Gammaproteobacteria	Cardiobacteriales	-	-	-
18	11	Bacteroidetes	Bacteroidia	Bacteroidales	Porphyromonadaceae	*Paludibacter*	-
19	11	Proteobacteria	Betaproteobacteria	Burkholderiales	Alcaligenaceae	-	-

20	10	Proteobacteria	Gammaproteobacteria	Pseudomonadales	Moraxellaceae	-	-
21	10	Proteobacteria	Gammaproteobacteria	Cardiobacteriales	-	-	-
22	10	Bacteroidetes	Flavobacteriia	Flavobacteriales	Flavobacteriaceae	*Tenacibaculum*	-
23	10	GN02	BD1-5	-	-	-	-
24	10	Firmicutes	Clostridia	Clostridiales	Lachnospiraceae	-	-

Supplemental Table 1: Taxonomic ID of ASVs present in ≥75% of samples with RBS-As detected. Only samples with ≥10 RBS-As visually confirmed were considered to bolster confidence in the assessment (e.g., that an RBS-B was not accidentally considered as an RBS-A). Out of 13 samples meeting this criterion, the number of samples in which any given ASV appears is shown, along with assigned taxonomic ID at the lowest possible taxonomic level. Dashes ("-") indicate that an ASV was not assigned at a given taxonomic level. Note that the ASV from family Alcaligenaceae has 100% sequence identity over 100% length to the 16S rRNA gene associated with the family Alcaligenaceae bin recovered from the mini-metagenomics experiment.

ASV	# samples	Phylum	Class	Order	Family	Genus	Species
1	11	Proteobacteria	Gammaproteobacteria	Pasteurellales	Pasteurellaceae	*Pasteurella*	*skyensis*
2	11	Proteobacteria	Gammaproteobacteria	Pseudomonadales	Moraxellaceae	-	-
3	11	Proteobacteria	Gammaproteobacteria	Cardiobacteriales	-	-	-
4	11	Bacteroidetes	Flavobacteriia	Flavobacteriales	Flavobacteriaceae	-	-
5	11	Fusobacteriota	Fusobacteriia	Fusobacteriales	Fusobacteriaceae	*Fusobacterium*	-
6	11	Proteobacteria	Gammaproteobacteria	Pasteurellales	Pasteurellaceae	*Pasteurella*	*skyensis*
7	11	Bacteroidetes	Flavobacteriia	Flavobacteriales	Flavobacteriaceae	*Tenacibaculum*	-
8	11	Firmicutes	Bacilli	Lactobacillales	Enterococcaceae	*Enterococcus*	-
9	10	Proteobacteria	Gammaproteobacteria	Pseudomonadales	Moraxellaceae	-	-
10	10	Proteobacteria	Gammaproteobacteria	Cardiobacteriales	-	-	-
11	10	Bacteroidetes	Bacteroidia	Bacteroidales	-	-	-
12	10	Bacteroidetes	Flavobacteriia	Flavobacteriales	Flavobacteriaceae	-	-
13	10	Bacteroidetes	Bacteroidia	Bacteroidales	Porphyromonadaceae	*Paludibacter*	-
14	10	Bacteroidetes	Flavobacteriia	Flavobacteriales	[Weeksellaceae]	-	-
15	9	Proteobacteria	Gammaproteobacteria	Pseudomonadales	Moraxellaceae	-	-
16	9	Proteobacteria	Epsilonproteobacteria	Campylobacterales	Campylobacteraceae	*Arcobacter*	-
17	9	Proteobacteria	Gammaproteobacteria	Cardiobacteriales	-	-	-

18	9	Bacteroidetes	Flavobacteriia	Flavobacteriales	Flavobacteriaceae	*Tenacibaculum*	-
19	9	Proteobacteria	Epsilonproteobacteria	Campylobacterales	Campylobacteraceae	-	-
20	9	Bacteroidetes	Flavobacteriia	Flavobacteriales	Flavobacteriaceae	-	-
21	9	Proteobacteria	Betaproteobacteria	Burkholderiales	Alcaligenaceae	-	-
22	9	Proteobacteria	Gammaproteobacteria	Cardiobacteriales	-	-	-
23	9	Proteobacteria	Epsilonproteobacteria	Campylobacterales	Campylobacteraceae	-	-
24	9	Bacteroidetes	Flavobacteriia	Flavobacteriales	Flavobacteriaceae	*Tenacibaculum*	-
25	9	Bacteroidetes	Bacteroidia	Bacteroidales	Bacteroidaceae	*Bacteroides*	*ovatus*
26	9	Firmicutes	Clostridia	Clostridiales	Lachnospiraceae	-	-

Supplemental Table 2: Taxonomic ID of ASVs present in ≥75% of samples with RBS-Bs detected. Only samples with ≥10 RBS-As visually confirmed were considered to bolster confidence in the assessment (e.g., that an RBS-A was not accidentally considered as an RBS-B). Out of 13 samples meeting this criterion, the number of samples in which any given ASV appears is shown, along with the assigned taxonomic ID at the lowest possible taxonomic level. Dashes (“-”) indicate that an ASV was not assigned at a given taxonomic level. Note that the ASV from family Alcaligenaceae has 100% sequence identity over 100% length to the 16S rRNA gene associated with the family Alcaligenaceae bin recovered from the mini-metagenomics experiment.

Bin ID	# scaffolds	Bin length	Longest scaffold	N50	Completeness (%)	Contamination (%)
1	272	1,941,293	26,105	6,813	1	0
2	56	846,971	68,899	18,366	49	6
3	25	288,673	27,320	10,819	14	0
4	45	732,195	52,808	24,418	34	0
5	36	434,317	31,157	15,055	18	0
6	93	1,026,923	32,958	12,480	59	0
7	16	116,224	14,175	7,023	2	0
8	15	127,029	15,569	8,224	0	0
9	12	92,216	12,253	7,606	0	0
10	121	1,176,422	35,727	9,462	31	2
11	56	1,591,011	224,748	40,556	76	1
12	204	3,272,710	104,534	23,600	90	40
13	187	1,783,018	24,881	10,073	58	10
14	12	137,833	25,347	14,892	7	0
15	21	171,018	13,722	99	0	0
16	43	712,931	90,478	16,606	42	1
17	89	801,486	40,325	9,084	12	0
18	68	1,679,601	97,578	33,569	83	15

Supplementary Table 3: Assembly statistics for bins recovered from the mini-metagenomics experiment. For each bin, the number of scaffolds, total length of all scaffolds, longest scaffold, N50, completeness (%), and contamination (%) are reported. Completeness and contamination were estimated based on the presence/absence of a broad set of marker genes using CheckM[2].

	RBS-A samples				Negative controls			
**Bin ID**	**1**	**2**	**3**	**4**	**1**	**2**	**3**	**4**
1	0.00	0.00	0.05	0.00	0.00	0.02	0.00	87.74
2	8.44	1.29	0.05	16.58	0.00	61.22	0.00	0.00
3	0.00	0.00	0.00	4.17	0.00	0.00	0.00	0.66
4	0.00	1.23	0.00	4.35	0.00	0.00	0.00	0.00
5	10.94	29.80	0.00	0.24	0.00	2.64	0.00	0.26
6	2.84	0.06	0.00	3.04	0.00	0.00	0.00	1.91
7	0.00	0.00	0.00	0.00	0.00	6.32	0.00	6.74
8	0.00	0.00	0.00	0.00	0.00	8.84	0.00	0.00
9	1.49	0.44	0.00	2.31	92.13	13.85	0.00	0.00
10	15.22	9.90	0.02	15.61	0.00	1.11	0.00	1.50
11	1.11	0.00	0.00	11.52	0.00	0.59	0.00	0.00
12	14.53	12.32	3.98	3.67	3.83	2.57	0.00	0.60
13	16.49	3.73	47.83	1.77	4.04	0.58	0.00	0.06
14	0.01	0.07	0.00	2.35	0.00	0.08	0.00	0.00
15	9.31	37.48	1.39	5.07	0.00	0.38	0.00	0.00
16	13.70	0.00	0.00	0.21	0.00	0.00	0.00	0.00
17	3.37	3.52	45.62	6.85	0.00	1.80	0.00	0.52
18	2.57	0.15	1.05	22.27	0.00	0.00	0.00	0.00
**# read pairs**	2,751,812	2,896,207	6,306,907	5,051,639	2,201,169	3,522,055	3,197,485	5,059,659
**# proper read pair alignments**	487,042	119,453	7,013	511,482	769	241,222	0	185,809

Supplementary Table 4: Relative abundance (%) of bins recovered in the mini-metagenomics experiment. We applied the following thresholds to aid in the evaluation of whether a given bin was likely to have been present in a sample, color-coded by relative abundance as follows: green, ≥ 5%; yellow, ≥ 1% and < 5%; orange, ≥ 0% and < 1%, red: 0%. The last two rows of the table show the total number of read

pairs per sample and the number of primary alignments with proper read pairs mapping to scaffolds ≥ 5 kb long per sample (only scaffolds ≥ 5 kb were binned; Methods).

REAGENT OR RESOURCE	SOURCE	IDENTIFIER
**Bacterial and eukaryotic strains**		
Caulobacter crescentus CB15N	Huang Lab	CB15N[3]
Escherichia coli MG1655	Coli Genetic Stock Center (CGSC)	CGSC #6300<br>F^-, lambda$^-$, *rph-1*<br>cogs.biology.yale.edu
Simonsiella muelleri ATCC29453	American Type Culture Collection (ATCC)	ATCC29453, https://www.atcc.org/products/29453
Skeletonema costatum LB 2308	UTEX Culture Collection of Algae	LB 2308, https://utex.org/products/utex-lb-2308
**Biological samples**		
Dolphin oral swabs	National Marine Mammal Foundation	https://www.nmmf.org/our-work/health-welfare/
**Chemicals**		
Agar	BD	Cat. #214530
Agarose	Invitrogen	Cat. #16-500-100
Bovine serum	Thermo Fisher Scientific	Cat. #16-170-078
Calcium chloride dihydrate	Sigma-Aldrich	Cat. #7902
DAPI	Invitrogen	Cat. #D1306
DEPC-treated water	Thermo Fisher Scientific	Cat. #AM9920
Dextran sulfate sodium salt	Fisher Scientific	Cat. #AAJ6360614
Dextrose	Thermo Fisher Scientific	Cat. #BP350500
Erdschreiber's Medium	UTEX Culture Collection of Algae	https://utex.org/
Ethanol, 200-proof	Sigma-Aldrich	Cat. #E7023-1L
FM-464 dye	Thermo Fisher Scientific	Cat. #T13320
Formaldehyde, 37%	Sigma-Aldrich	Cat. #47608-1L-F

Formamide	Sigma-Aldrich	Cat. #F9037-100ML
Glycerol	Sigma-Aldrich	Cat. #G5516-500ML
Hemin chloride	MP Biomedicals	Cat. #MP21988202
L-arginine, reagent grade, >98%	Sigma-Aldrich	Cat. #A5006-500G
LB broth	RPI research products	Cat. #L24066-1000.0
Magnesium chloride hexahydrate	Sigma-Aldrich	Cat. #M2393
Menadione (Vitamin K)	Sigma-Aldrich	Cat. #M5425-25G
Mucin (gastric)	Pfaltz & Bauer	Cat. #M32610100g
N-acetyl muramic acid	Sigma-Aldrich	Cat. #A3007-100MG
PBS, 10X	Thermo Fisher Scientific	Cat. #AM9624
PBS, 1X	Thermo Fisher Scientific	Cat. #10010049
Potassium chloride	Fisher Scientific	Cat. #P330-500
Potassium phosphate monobasic	Fisher Scientific	Cat. # BP363-500
Protease peptone	Sigma-Aldrich	Cat. #82450
Sheep blood	Thermo Fisher Scientific	Cat. #R54016
Sodium bicarbonate	Sigma-Aldrich	Cat. #S5761
Sodium chloride	Fisher Scientific	Cat. #S271-500
SSC, 20X	Fisher Scientific	Cat. #BP1325-1
Sucrose	Thermo Fisher Scientific	Cat. #AAA1558336
Tryptic soy broth	Thermo Fisher	Cat. #DF0370-17-3
Trypticase peptone	BD	Cat. #211921
Urea	Sigma-Aldrich	Cat. #U5378
Yeast extract	Thermo Scientific	Cat. #212750
**Critical Commercial Assays**		

DNeasy UltraClean 96 Microbial Kit	Qiagen	Cat. #10196-4
Gram Staining Kit	Sigma Aldrich	Cat. #77730-1KT-F
Kapa Hyper Prep Kit	Kapa Biosystems	Cat. #KK8504
Macherey-Nagel NucleoSpin Gel and PCR Clean-up Mini Kit	Fisher	Cat. #740609
Platinum II HotStart PCR Master Mix	Thermo Fisher	Cat. #14000013
Repli-g single cell kit	Qiagen	Cat. #150343
Zymo Clean and Concentrate Spin Column	Zymo Research Corporation	Cat. #D4013
**Deposited Data**		
NCBI non-redundant nucleotide database	https://www.ncbi.nlm.nih.gov/protein/	https://www.ncbi.nlm.nih.gov/nucleotide/
NCBI non-redundant protein database	https://www.ncbi.nlm.nih.gov/protein/	https://www.ncbi.nlm.nih.gov/protein/
NCBI Taxonomy Database	https://www.ncbi.nlm.nih.gov/taxonomy	https://www.ncbi.nlm.nih.gov/taxonomy; See Supplementary Figures 4 and 5 for accession numbers of 16S rRNA gene (n=77) and ribosomal protein S3 sequences (n=63) used
Pfam database accessed August 2022; alignment for AmiC2	ref[4]	http://pfam.xfam.org/; accession PF01520
Pfam database accessed March 2019; alignments for ribosomal proteins L2, L3, L4, L5, L6, L14, L15, L16, L18, L22, L24, S3, S8, S10, S17, S19	ref[4]	http://pfam.xfam.org/; accessions PF00181, PF00297, PF00573, PF00281, PF00347, PF00238, PF00828, PF00252, PF00861, PF00237, PF17136, PF00189, PF00410, PF00338, PF00366, PF00203
SILVA SSU Database, release 138.1	ref[5]	https://www.arb-silva.de/documentation/release-138/
**Oligonucleotides**		
515F forward primer	Integrated DNA Technologies	https://www.idtdna.com/pages

806rB reverse primer	Integrated DNA Technologies	https://www.idtdna.com/pages
Bact-338+AlexaFluor-488	Integrated DNA Technologies	https://www.idtdna.com/pages
BET42a+AlexaFluor-594	Integrated DNA Technologies	https://www.idtdna.com/pages
Euk-1209+AlexaFluor-660	Integrated DNA Technologies	https://www.idtdna.com/pages
GAM42a+AlexaFluor-488	Integrated DNA Technologies	https://www.idtdna.com/pages
nonEUC+Cy5	Integrated DNA Technologies	https://www.idtdna.com/pages
**Software and Algorithms**		
Bcl2Fastq v. 2	Illumina	https://www.illumina.com/
BLAST v. 2.2.30	ref[1]	https://blast.ncbi.nlm.nih.gov/Blast.cgi
Bowtie2 v. 2.2.4	ref[6]	https://github.com/BenLangmead/bowtie2
CheckM v. 1.0.7	ref[2]	https://github.com/Ecogenomics/CheckM
Clustal Omega v. 1.2.4	ref[7,8]	https://www.ebi.ac.uk/Tools/msa/clustalo/
DADA2 v. 1.16.0	ref[9]	https://github.com/benjjneb/dada2
Databionics ESOM Tools software	ref[10]	http://databionic-esom.sourceforge.net
EMAN2 v. 2.39	ref[11]	https://blake.bcm.edu/emanwiki/EMAN2
Fiji v. 2.0.0	ref[12]	https://fiji.sc/
HMMER suite v. 3.1b2	ref[13]	http://hmmer.org/
IMOD v. 4.12.9	ref[14]	https://bio3d.colorado.edu/imod/
iTOL v. 6	ref[15]	https://itol.embl.de/
Phyloseq v. 1.28.0	ref[16]	https://joey711.github.io/phyloseq/
PhyML v. 3.1	ref[17]	http://www.atgc-montpellier.fr/phyml/
Prodigal v. 2.6.2	ref[18]	https://github.com/hyattpd/Prodigal
Samtools v. 1.6	ref[19]	http://samtools.sourceforge.net/

SerialEM v. 3.8	ref[20]	https://bio3d.colorado.edu/SerialEM/
SINA v. 1.2.11	ref[21]	https://www.arb-silva.de/aligner/
Smart Model Selection v. 2.0	ref[22]	http://www.atgc-montpellier.fr/sms/
SPAdes v. 3.11.1	ref[23]	http://cab.spbu.ru/software/spades/
Strain Library Imaging Protocol, 2016 implementation	ref[24]	https://www.nature.com/articles/nprot.2016.181
UCSF Chimera v. 1.16	ref[25]	https://www.cgl.ucsf.edu/chimera/
**Other**		
15 nm gold fiducial beads	Sigma Aldrich	Cat. #777137
300 kV TEM with energy filter	Thermo Fisher Scientific (FEI Company)	Titan Krios G3
300 kV TEM without energy filter	Thermo Fisher Scientific (FEI Company)	Titan Krios G4
Anaerobic chamber	COY	https://coylab.com/products/anaerobic-chambers/
Aquilous Cryo-FIB	Thermo Fisher Scientific	https://www.thermofisher.com/order/catalog/product/AQUILOSFIB
Catch-All sample collection swabs	Epicenter	Cat. #QEC091H
Eclipse Ti microscope	Nikon	https://www.microscope.healthcare.nikon.com/products/inverted-microscopes/eclipse-ti-series
Energy filter	Gatan	BioQuantum GIF, model #967
Grid vitrification plunge freezing device	Leica	EM-GP
Holey carbon TEM grids, R 2/1 200 mesh, copper	Quantifoil	Cat. #Q2100CR1
London Finder TEM grids (lettered), R 2/2, LF, 200 Mesh, Gold	Quantifoil	Cat. #LFH2100AR2
Micromanipulator	Eppendorf	Transferman micromanipulator with SAS-10 microinjector

MiSeq 2x250 nt P2 V2	Illumina	https://www.illumina.com/
Olympus microscope	Olympus Life Science Solutions	IX70 inverted microscope
Polar Body Biopsy Micropipettes	Cooper Surgical	https://fertility.coopersurgical.com/micropipettes/biopsy-micropipettes/
TEM direct electron detector	Gatan	K2 Summit

Supplementary Table 5: Key reagents and resources used in this study. They are organized into functional sections (bacterial and eukaryotic strains; biological samples; chemicals; critical commercial assays; deposited data; oligonucleotides; software and algorithms; other) and listed in alphabetical order within each section. For each reagent or resource, their source and an identifier are provided.

TEM instrument	Data collection type	Pixel size (Å/pixel)	Corresponding figure(s)	Applied defocus (µm)	Cumulative dose ($e^-/Å^2$)
Titan Krios G3, 300 keV, energy filter at 20-eV slit width, K2 detector	Tilt series	3.48	Figure 7c	-6	120
	2D montage maps and/or images	1.06	Figure 7a<br>Figure S7d	-5	80
		3.48	Figure 7b<br>Figure 5d	-5	
Titan Krios G2, 300 keV, no energy filter, K2 detector	Tilt series	3.75	Figure 6b	-6	120
		7.5	Figure 6a	-6	
	2D montage maps and/or images	1.43	Figure 5a<br>Figure 5e	-5	80
		3.75	Figure 5b	-5	
		14	Figure 5c	-50	

Supplementary Table 6: CryoET/EM imaging parameters. Samples were loaded into one of two cryo-transmission electron microscopes. Two-dimensional images and montages were aligned, and dose-fractionated movies were acquired in counting mode. Tilt series were collected bidirectionally from -21°, through a range from -60° to +60° in 3° increments.

SUPPLEMENTARY REFERENCES

1. Altschul, S. F., Gish, W., Miller, W., Myers, E. W. & Lipman, D. J. Basic local alignment search tool. *J Mol Biol* **215**, 403-410 (1990). https://doi.org:10.1016/S0022-2836(05)80360-2
2. Parks, D. H., Imelfort, M., Skennerton, C. T., Hugenholtz, P. & Tyson, G. W. CheckM: assessing the quality of microbial genomes recovered from isolates, single cells, and metagenomes. *Genome Res* **25**, 1043-1055 (2015). https://doi.org:10.1101/gr.186072.114
3. Evinger, M. & Agabian, N. Envelope-associated nucleoid from *Caulobacter crescentus* stalked and swarmer cells. *J Bacteriol* **132**: 294–301 (1977).
4. Finn, R. D. *et al.* The Pfam protein families database: towards a more sustainable future. *Nucleic Acids Res* **44**, D279-285 (2016). https://doi.org:10.1093/nar/gkv1344
5. Yilmaz, P. *et al.* The SILVA and "all-species living tree project (LTP)" taxonomic frameworks. *Nucleic acids research* **42**, D643-D648 (2014).
6. Langmead, B. & Salzberg, S. L. Fast gapped-read alignment with Bowtie 2. *Nat Methods* **9**, 357-359 (2012). https://doi.org:10.1038/nmeth.1923
7. Goujon, M. *et al.* A new bioinformatics analysis tools framework at EMBL–EBI. *Nucleic acids research* **38**, W695-W699 (2010).
8. Sievers, F. *et al.* Fast, scalable generation of high-quality protein multiple sequence alignments using Clustal Omega. *Molecular systems biology* **7**, 539 (2011).
9. Callahan, B. J. *et al.* DADA2: High-resolution sample inference from Illumina amplicon data. *Nat Methods* **13**, 581-583 (2016). https://doi.org:10.1038/nmeth.3869
10. Ultsch, A. & Mörchen, F. *ESOM-Maps: tools for clustering, visualization, and classification with Emergent SOM*. Vol. 46 (Univ., 2005).
11. Tang, G. *et al.* EMAN2: an extensible image processing suite for electron microscopy. *J Struct Biol* **157**, 38-46 (2007). https://doi.org:10.1016/j.jsb.2006.05.009
12. Schindelin, J. *et al.* Fiji: an open-source platform for biological-image analysis. *Nat Methods* **9**, 676-682 (2012). https://doi.org:10.1038/nmeth.2019

13. Eddy, S. R. Accelerated Profile HMM Searches. *PLoS Comput Biol* **7**, e1002195 (2011). https://doi.org:10.1371/journal.pcbi.1002195
14. Kremer, J. R., Mastronarde, D. N. & McIntosh, J. R. Computer visualization of three-dimensional image data using IMOD. *J Struct Biol* **116**, 71-76 (1996). https://doi.org:10.1006/jsbi.1996.0013
15. Letunic, I. & Bork, P. Interactive Tree Of Life (iTOL) v5: an online tool for phylogenetic tree display and annotation. *Nucleic acids research* **49**, W293-W296 (2021).
16. McMurdie, P. J. & Holmes, S. phyloseq: an R package for reproducible interactive analysis and graphics of microbiome census data. *PLoS One* **8**, e61217 (2013). https://doi.org:10.1371/journal.pone.0061217
17. Guindon, S. *et al.* New algorithms and methods to estimate maximum-likelihood phylogenies: assessing the performance of PhyML 3.0. *Systematic biology* **59**, 307-321 (2010).
18. Hyatt, D. *et al.* Prodigal: prokaryotic gene recognition and translation initiation site identification. *BMC Bioinformatics* **11**, 119 (2010). https://doi.org:10.1186/1471-2105-11-119
19. Li, H. *et al.* The Sequence Alignment/Map format and SAMtools. *Bioinformatics* **25**, 2078-2079 (2009). https://doi.org:10.1093/bioinformatics/btp352
20. Mastronarde, D. N. SerialEM: a program for automated tilt series acquisition on Tecnai microscopes using prediction of specimen position. *Microscopy and Microanalysis* **9**, 1182-1183 (2003).
21. Pruesse, E., Peplies, J. & Glöckner, F. O. SINA: accurate high-throughput multiple sequence alignment of ribosomal RNA genes. *Bioinformatics* **28**, 1823-1829 (2012).
22. Lefort, V., Longueville, J.-E. & Gascuel, O. SMS: smart model selection in PhyML. *Molecular biology and evolution* **34**, 2422-2424 (2017).
23. Bankevich, A. *et al.* SPAdes: a new genome assembly algorithm and its applications to single-cell sequencing. *J Comput Biol* **19**, 455-477 (2012). https://doi.org:10.1089/cmb.2012.0021
24. Shi, H., Colavin, A., Lee, T. K. & Huang, K. C. Strain Library Imaging Protocol for high-throughput, automated single-cell microscopy of large bacterial collections

arrayed on multiwell plates. *Nat Protoc* **12**, 429-438 (2017). https://doi.org:10.1038/nprot.2016.181
25. Pettersen, E. F. *et al.* UCSF Chimera--a visualization system for exploratory research and analysis. *J Comput Chem* **25**, 1605-1612 (2004). https://doi.org:10.1002/jcc.20084
