# Supplementary material for: Previously uncharacterized rectangular bacterial structures in the dolphin mouth
Source: Nat Commun. 2023 Apr 13;14:2098. doi: 10.1038/s41467-023-37638-y (PMC10102025; doi:10.1038/s41467-023-37638-y)
Supplement: Supplementary file 3 — Description of Additional Supplementary Files [file 41467_2023_37638_MOESM3_ESM.pdf]

Description of Additional Supplementary Files:

Supplementary Data 1. Prevalence of RBSs in dolphin oral samples. 73 samples from eight dolphins were evaluated for the presence of RBSs using SLIP (Shi et al., 2017) via phase contrast microscopy. The order of magnitude of RBS counts for each morphology per sample is shown here; * denotes 1-9 RBSs of a given morphotype detected, ** denotes 10-99, and *** denotes 100 or more (maximum identified was ~366 in a single sample). Samples collected prior to 2022 were surveyed using 226 fields of view (FOVs) and underwent amplicon sequencing, while those collected in 2022 were surveyed using 100 FOVs and did not undergo sequencing. RBS-As and RBS-Bs were each detected in 39/73 samples (53%) and 42/73 samples (58%), respectively. RBS-As were detected in samples from 7/8 dolphins, while RBS-Bs were detected in samples from 6/8 dolphins."

Supplementary Movie 1. RBS-As are not cylindrical. An RBS-A partially stuck to a Petri dish fluttered as suction was applied by and released from a micropipette. Note that this video was captured solely to show the morphology of the RBS-As. Debris was inevitably captured as a result of applying sufficient suction for the RBS-A to flutter on its side. No RBS-As were collected for the minimetagenomics experiment during the recording of this video or afterwards using this micropipette.

Supplementary Movie 2. CryoET reveals the 3D architecture of RBS-A components, example 1. Yellow, pilus-like appendages; blue, inner membrane; purple, S-layer-like structure; green, outer membrane; red, matrix. Scale bar: 100 nm.

Supplementary Movie 3. CryoET reveals the 3D architecture of RBS-A components, example 2. Yellow, pilus-like appendages; blue, inner membrane; purple, S-layer-like structure; green, outer membrane; red, matrix. Scale bar: 100 nm.
