# Supplementary material for: Previously uncharacterized rectangular bacterial structures in the dolphin mouth
Source: Nat Commun. 2023 Apr 13;14:2098. doi: 10.1038/s41467-023-37638-y (PMC10102025; doi:10.1038/s41467-023-37638-y)
Supplement: Supplementary file 8 — Reporting Summary [file 41467_2023_37638_MOESM8_ESM.pdf]

nature portfolio

Corresponding author(s): David Relman

Last updated by author(s): Mar 10, 2023

Reporting Summary

Nature Portfolio wishes to improve the reproducibility of the work that we publish. This form provides structure for consistency and transparency in reporting. For further information on Nature Portfolio policies, see our Editorial Policies and the Editorial Policy Checklist.

Statistics

For all statistical analyses, confirm that the following items are present in the figure legend, table legend, main text, or Methods section.

n/a	Confirmed	
☐	☒	The exact sample size (n) for each experimental group/condition, given as a discrete number and unit of measurement
☐	☒	A statement on whether measurements were taken from distinct samples or whether the same sample was measured repeatedly
☐	☒	The statistical test(s) used AND whether they are one- or two-sided<br>*Only common tests should be described solely by name; describe more complex techniques in the Methods section.*
☒	☐	A description of all covariates tested
☐	☒	A description of any assumptions or corrections, such as tests of normality and adjustment for multiple comparisons
☐	☒	A full description of the statistical parameters including central tendency (e.g. means) or other basic estimates (e.g. regression coefficient) AND variation (e.g. standard deviation) or associated estimates of uncertainty (e.g. confidence intervals)
☒	☐	For null hypothesis testing, the test statistic (e.g. F, t, r) with confidence intervals, effect sizes, degrees of freedom and P value noted<br>*Give P values as exact values whenever suitable.*
☒	☐	For Bayesian analysis, information on the choice of priors and Markov chain Monte Carlo settings
☒	☐	For hierarchical and complex designs, identification of the appropriate level for tests and full reporting of outcomes
☒	☐	Estimates of effect sizes (e.g. Cohen's d, Pearson's r), indicating how they were calculated

Our web collection on statistics for biologists contains articles on many of the points above.

Software and code

Policy information about availability of computer code

Data collection	CryoEM/ET data was collected using SerialEM version 3.8<br>High-throughput light microscopy imaging data was collected using the Strain Library Imaging Protocol (2016 implementation, no version number).
Data analysis	CryoEM/ET data was analyzed using IMOD v. 4.12.9, EMAN2 v. 2.39, UCSF Chimera v. 1.16<br>Light microscopy data was analyzed using FIJI v. 2.0.0<br>16S rRNA gene amplicon analysis was carried out using Bcl2Fastq v. 2, DADA2 v. 1.16.0, phyloseq v. 1.28.0<br>The mini-metagenomics data was analyzed using SPAdes v. 3.11.1, Prodigal v. 2.6.2, bowtie2 v. 2.2.4, samtools v. 1.6. HMMER suite v. 3.1b2, Databionics ESOM Tools software v. 1.1, CheckM v. 1.0.7, BLAST v. 2.2.30, CheckM v. 1.0.7, SINA v. 1.2.11, Clustal Omega v. 1.2.4, PhyML v. 3.1, Smart Model Selection v. 2.0, iTOL v. 6

For manuscripts utilizing custom algorithms or software that are central to the research but not yet described in published literature, software must be made available to editors and reviewers. We strongly encourage code deposition in a community repository (e.g. GitHub). See the Nature Portfolio guidelines for submitting code & software for further information.

Data

Policy information about availability of data

All manuscripts must include a data availability statement. This statement should provide the following information, where applicable:

- Accession codes, unique identifiers, or web links for publicly available datasets
- A description of any restrictions on data availability
- For clinical datasets or third party data, please ensure that the statement adheres to our policy

Data generated in-house are available as follows:
Sequencing data for this project are available through NCBI BioProject PRJNA174530. Raw reads for the amplicon survey were deposited to SRA and are associated with BioSamples SAMN32739817-69. Those for the spike-in experiment are associated with BioSamples SAMN32869723-5. Raw reads for the single cell genomics experiment were also deposited to SRA; the captured RBSs and negative controls are physically derived from a single oral swab represented by BioSample SAMN19012476, while the reads each from the eight experimental replicates (four RBSs, four negative controls) are each individually associated with BioSamples SAMN19022663-SAMN19022670. The co-assembly of scaffolds ≥5kb in length from the single cell genomics experiment was deposited as a Whole Genome Shotgun project at DDBJ/ENA/GenBank under the accession JAHCSF000000000, following the removal of human-derived sequences. The version described in this paper is version JAHCSF010000000. Genome bins 2-5 and 7-18 from the single cell genomics experiment have been deposited as a Whole Genome Shotgun project at DDBJ/ENA/GenBank under accessions JAGYHI000000000-JAGYHX000000000. The versions described in this paper are versions JAGYHI010000000-JAGYHX010000000. Genome bin 1 (human) was not deposited. Scaffolds for genome bin6 (<100,000 nucleotides) were deposited as a non-genome GenBank submission under accession numbers MZ126582-MZ126593.

We used data from the following public sources:
SILVA SSU database v. 138.1. Sequences in the database were used as a reference for assigning taxonomic identities to ASVs.
the NCBI nr/nt database, nr database, and taxonomy database all accessed August 2022. These databases were used to identified 16S rRNA gene sequences and ribosomal protein S3 sequence for representatives from genera of the family Alcaligenaceae. The accession numbers for identified 16S rRNA gene sequences (n=77) and ribosomal protein S3 sequences (n=63) are available in Supplementary Figures 4 and 5, respectively.
Pfam database accessed March 2019 and August 2022. Pfam alignment PF01520 was used to search genome bins for AmiC2 proteins (accessed August 2022). The following Pfam alignments were used to search for 16 bacterial single copy genes (accessed March 2019): PF00181, PF00297, PF00573, PF00281, PF00347, PF00238, PF00828, PF00252, PF00861, PF00237, PF17136, PF00189, PF00410, PF00338, PF00366, PF00203.

Human research participants

Policy information about studies involving human research participants and Sex and Gender in Research.

Reporting on sex and gender	Our study does not involve human research participants
Population characteristics	Our study does not involve human research participants
Recruitment	Our study does not involve human research participants
Ethics oversight	Our study does not involve human research participants

Note that full information on the approval of the study protocol must also be provided in the manuscript.

Field-specific reporting

Please select the one below that is the best fit for your research. If you are not sure, read the appropriate sections before making your selection.

☒ Life sciences ☐ Behavioural & social sciences ☐ Ecological, evolutionary & environmental sciences

For a reference copy of the document with all sections, see nature.com/documents/nr-reporting-summary-flat.pdf

Life sciences study design

All studies must disclose on these points even when the disclosure is negative.

Sample size	Sample size calculations were not performed. Sample sizes were chosen based on the maximum number of samples that were logistically feasible to collect from the mouths of dolphins. Sinces samples typically contained multiple RBSs, this did not limit efforts to characterize individual RBSs within samples. No treatments or different conditions were analyzed.
Data exclusions	No data were excluded from the study
Replication	We did not attempt to replicate experiments performed in this study. No treatments were performed. No different conditions were analyzed.
Randomization	This was a descriptive study. There was no experimental group.
Blinding	During analyses related to counting the number of RBSs of each morphotype in distinct oral sites (palatal, gingival, buccal), researchers were

blinded as to which sample was from which oral site. No other blinding was performed, as the focus of this study was describing morphological features of RBSs and attempting to taxonomically identify the RBSs.

Reporting for specific materials, systems and methods

We require information from authors about some types of materials, experimental systems and methods used in many studies. Here, indicate whether each material, system or method listed is relevant to your study. If you are not sure if a list item applies to your research, read the appropriate section before selecting a response.

Materials & experimental systems

n/a	Involved in the study
☒	☐ Antibodies
☒	☐ Eukaryotic cell lines
☒	☐ Palaeontology and archaeology
☒	☐ Animals and other organisms
☒	☐ Clinical data
☒	☐ Dual use research of concern

Methods

n/a	Involved in the study
☒	☐ ChIP-seq
☒	☐ Flow cytometry
☒	☐ MRI-based neuroimaging
